# Admission hypoxia-inducible factor 1α levels and in-hospital mortality in patients with acute decompensated heart failure

**DOI:** 10.1186/s12872-015-0073-6

**Published:** 2015-07-30

**Authors:** Gang Li, Wei-hua Lu, Xiao-wei Wu, Jian Cheng, Rong Ai, Zi-hua Zhou, Zhong-zhi Tang

**Affiliations:** Emergency Department, Wuhan General Hospital of Guangzhou Military Command, Wuhan, 430074 China; Department of Thoracic Surgery, TongJi Hospital, TongJi Medical College, Huazhong University of Science and Technology, Wuhan, China; College of Foreign Language, Huazhong Agriculture University, Wuhan, China; Institute of Cardiology, Union Hospital, Tongji Medical College, Huazhong University of Science & Technology, Wuhan, China

**Keywords:** Hypoxia-inducible factor 1α, Acute decompensated heart failure, N-terminal–pro-brain natriuretic peptide, In-hospital mortality

## Abstract

**Background:**

Hypoxia-inducible factor 1 (HIF-1) is a critical regulator for cellular oxygen balance. Myocardial hypoxia can induce the increased expression of HIF-1α. Our goals were to evaluate the value of HIF-1α in predicting death of patients with acute decompensated heart failure (ADHF) and describe the in vivo relationship between serum HIF-1α and N-terminal–pro-brain natriuretic peptide (NT-proBNP) levels.

**Method:**

We included 296 patients who were consecutively admitted to the emergency department for ADHF. The primary end point was in-hospital death. The patients were categorized as HF*r*EF (patients with reduced systolic function) and HF*p*EF (patients with preserved systolic function) groups.

**Results:**

In our patients, the median admission HIF-1α level was 2.95 ± 0.85 ng/ml. The HIF-1α level was elevated significantly in HF*r*EF patients and deceased patients compared with HF*p*EF patients and patients who survived. The HIF-1α level was positively correlated with NT-proBNP and cardiac troponin T levels, and negatively correlated with left ventricular ejection fraction and systolic blood pressure. Kaplan–Meier curves revealed a significant increase in in-hospital mortality in ADHF patients with higher HIF-1α levels. Multivariable Cox regression analysis showed that HIF-1α levels were not correlated with the short-term prognosis of ADHF patients.

**Conclusions:**

This is the first study to evaluate the circulating levels of HIF-1α in ADHF patients. Serum HIF-1α levels may reflect a serious state in patients with ADHF. Due to the limitations of the study, serum HIF-1α levels were not correlated with the in-hospital mortality based on regression analysis. Further studies are needed to demonstrate the diagnostic and/or prognostic role of HIF-1α as a risk biomarker in patients with ADHF.

## Background

Heart failure is a common cardiovascular disease. Cardiac dysfunction may induce inadequate tissue perfusion, which leads to hypoxic ischemia of many organs. When myocardial cells are hypoxic, glucose serves as the substrate for glycolysis and fatty acids are converted to lipids [1, 2]; however, hypoxic myocardial cells cannot produce sufficient adenosine triphosphate to maintain cardiac function. The loss in efficacy of cardiac function is characterized by a decrease in the ejection fraction, an increase in left ventricular end-diastolic diameter (LVEDD), and the emergence of clinical symptoms and signs of heart failure [3, 4]. Intracellular tissue adaptation to hypoxia is mediated by hypoxia-inducible factor 1 (HIF-1), which is a key mediator in the transformation from oxidation to glycolysis [5]. In addition, the conversion from fatty acids to lipids is also mediated by HIF-1 [6].

HIF-1 is a transcription factor and a critical regulator for cellular oxygen balance. HIF-1 is a heterodimer comprised of two sub-units (α and β). HIF-1α controls oxygen transfer by regulating angiogenesis and vascular remodeling [7]. Moreover, the utility of oxygen is also controlled by HIF-1 via regulation of glucose metabolism and redox equilibrium [8]. A previous study showed that the expression of HIF-1α is increased significantly in patients with myocardial hypoxia [9]. As a transcription factor, HIF-1α mediates important physiologic responses during hypoxia by regulating downstream target genes to make the body produce compensatory adaptations to myocardial hypoxia. Animal models have shown that HIF-1α has a critical protective effect on the pathophysiology underlying ischemic heart disease [7, 10, 11] and pressure overload heart failure [12].

B-type natriuretic peptide (BNP) is primarily synthesized in ventricular cells [13, 14] and plays an important role in maintaining fluid balance and adjusting blood pressure [15]. Especially in patients with acute decompensated heart failure (ADHF), an elevated admission BNP level is a significant predictor of in-hospital mortality [16–18]. The synthesis of BNP is directly caused by hypoxia mediated through a HIF-1α-independent mechanism not influenced by hemodynamics or stimulation of neurohormones based on an in vitro ventricular myocyte model system [19]. BNP appears to be part of the protective program directed by HIF-1α in response to oxygen deprivation [20]. Cumulative experimental data have clearly shown that hypoxia is an independent factor regulating the natriuretic peptide system [21]. Additionally, hypoxia-response elements have been characterized from the promoter sequences of the ANP and BNP genes [20, 22, 23]; however, as an upstream regulation factor of BNP, the role of HIF-1α in ADHF patients has not been studied. In the current study we investigated whether or not the admission HIF-1α level predicted in-hospital mortality of ADHF patients and clarified the in vivo relationship between N-terminal–pro-brain natriuretic peptide (NT-proBNP).

## Methods

### Patients

Two hundred ninety-six patients with typical heart failure symptoms and signs were enrolled in the investigation conducted in conjunction with the Emergency Department. The criteria recommended by the most recent guidelines of the European Cardiology Society (ESC) and the American College of Cardiology Foundation/ American Heart Association (ACCF/AHA) were adopted for the classification of patients enrolled in this study [24, 25]. Specifically, the inclusion criteria were as follows: patients had presented within the previous 24 h with ADHF; diagnosed on the basis of the presence of at least one symptom (dyspnea, orthopnea, or edema) and one sign (rales, peripheral edema, ascites, or pulmonary vascular congestion on chest radiography) of heart failure; NYHA functional class III or IV, with an acute exacerbation of symptoms of at least 1 class; evidence of systolic and/or diastolic dysfunction by echocardiography; > 18 years of age; and NT-proBNP ≥ 1800 pg/ml. The NT-proBNP cut-off value of 1800 pg/ml was selected to increase specificity. The exclusion criteria were as follows: patients with tumors, unstable angina, or recent acute myocardial infarction, current or past dialysis; and patients in shock or without the company of a lineal relative and could not sign the informed consent. This study complied with the Helsinki Treaty. All participants signed the written informed consent form. Our study was approved by the Institutional Ethics Committee of Wuhan General Hospital of Guangzhou Military Command.

### Biochemical measurements

After patients signed the informed consent, blood samples were obtained immediately. The blood samples were centrifuged at 3000 rpm at 4 °C for 15 min. The supernatants were decanted and frozen at −80 °C until assayed. Serum creatinine, uric acid (UC), blood urea nitrogen (BUN), and high-sensitive C-reactive protein (hs-CRP) levels were determined in the hospital laboratory using standard methods. Enzyme-linked immunosorbent assay (ELISA) kits were used to measure serum levels of HIF-1α (Abnova, Taiwan). Intra- and inter-assay coefficients of variation for HIF-1α were 4.2 % and 7.6 %, respectively. The lower limit of detection (LOD) was 0.041 ng/ml and the reference value interval was 0.078–5.0 ng/ml. The plasma levels of NT-proBNP were determined using the Elecsys proBNP assay (Roche Diagnostics, Basel, Switzerland) [26]. The intra-assay coefficients of variation are 2.4 % and 1.8 % at 355 and 4962 pg/ml, respectively, and the respective inter-assay coefficients of variation were 2.9 % and 2.3 %, respectively. We measured TnT levels using a commercial one-step enzyme immunoassay (EIA) based on electrochemiluminescence technology (fourth-generation TnT, Elecsys 2010; Roche Diagnostics). The lower limit of detection of this assay was 10 ng/L, with a recommended diagnostic threshold of 30 ng/L. At this concentration, TnT concentrations can be measured with a coefficient of variation (CV) of < 10 % [27].

### Echocardiography

Patients were simultaneously evaluated with two-dimensional echocardiogram using standard views and protocols [28]. Pulsed-wave Doppler echocardiography was performed by an experienced cardiologist with a Hewlett Packard Sonos 1000 ultrasound system and a 2.5-MHz transducer (Palo Alto, California, USA). The main parameter which was evaluated was the left ventricular ejection fraction (LVEF). The patients were categorized as HF*r*EF (patients with reduced systolic function [LVEF ≤ 40 %]) and HF*p*EF (patients with preserved systolic function [LVEF > 40 %]) groups [25]. Doppler echocardiographic indices (e’, E/e’ ratio, left atrial volume index and LV mass index) to measure diastolic dysfunction were evaluated as recommended by ESC and ACCF/AHA guidelines [24, 25]. The diagnosis of HFpEF in our patients requires 3 conditions to be satisfied: typical symptoms and signs of HF, normal LVEF and LV not dilated, left ventricular diastolic dysfunction.

### Statistical analysis

SPSS version 18.0 (SPSS Inc, Chicago, Illinois) statistical software was used for statistical analysis. Continuous variables in a normal distribution were compared using the Student’s *t*-test and ANOVA. Categorical variables were analyzed using the chi-squared test. Pearson correlation analysis was used to assess the correlation between HIF-1α level and other cardiovascular disease risk factors. In order to clear whether the HIF-1α levels can predict the in-hospital mortality of ADHF patients, multivariate logistic regression models was used for the analysis. In the logistic regression models, we performed log-transformation of NT-proBNP and TnT. Survival curves were estimated according to the Kaplan-Meier method and compared by the log-rank test. Additionally, receiver operating characteristic analysis was performed to determine the cut-off value for HIF-1α in predicting type of ADHF with high sensitivity and specificity. *P* value <0.05 was considered statistical significant.

## Results

A total of 296 ADHF patients were enrolled between January 2011 and March 2014 at 4 clinical sites (Wuhan, China). Meanwhile, we also enrolled 52 healthy volunteers (mean age: 43.8 ± 18.5; male: 67.3 %) as control group. Of these ADHF patients, 21(7.1 %) died in hospital. In Table 1, baseline characteristics of patients are described. Among them, 195(65.9 %) cases were male. The mean age was 73.5 ± 10.4 years old. There were 188 (63.5 %) patients with HF*r*EF (median LVEF 35 %) and 108 (36.5 %) patients with HF*p*EF (median LVEF 52 %).

**Table 1:** Clinical and laboratory data for 296 patients with acute decompensated heart failure

Variable	HF*p*EF	HF*r*EF	*P* Value
	(*n* = 108)	(*n* = 188)	
Age (years)	74.0 ± 10.2	73.3 ± 10.5	0.599
Male *n*, (%)	77(71.3 %)	118(62.8 %)	0.161
History			
Coronary artery disease *n*, (%)	58(53.7 %)	96(51.25 %)	0.717
Hypertension *n*, (%)	79(73.1 %)	132(70.2 %)	0.689
Previous heart failure *n*, (%)	62(57.4 %)	90(47.9 %)	0.118
Diabetes mellitus *n*, (%)	49(45.4 %)	100(53.2 %)	0.227
COPD/asthma *n*, (%)	27(25 %)	40(21.3 %)	0.474
Atrial fibrillation *n*, (%)	35(32.4 %)	55(29.3 %)	0.601
Chronic renal insufficiency *n*, (%)	22 (20.4 %)	44 (23.4 %)	0.883
Cardiac valvular disease *n*, (%)	30(27.8 %)	39(20.7 %)	0.199
Intravenous medications during hospitalization			
Diuretics *n*, (%)	97(89.8 %)	172(91.5 %)	0.677
Cedilanid *n*, (%)	38(35.2 %)	89(47.3 %)	0.051
Nitroglycerin *n*, (%)	10(9.3 %)	59(31.4 %)	0.522
Nitroprusside *n*, (%)	10(9.3 %)	23(12.2 %)	0.565
Oral medications during hospitalization			
ACE inhibitors *n*, (%)	61(56.5 %)	121(64.4 %)	0.215
ARB *n*, (%)	23(21.3 %)	30(16.0 %)	0.272
Beta-blocker *n*, (%)	73(67.6 %)	141(75 %)	0.180
Calcium channel blocker *n*, (%)	19(17.6 %)	42(22.3 %)	0.372
Digoxin *n*, (%)	47(43.5 %)	110(58.5 %)	0.016
Diuretics *n*, (%)	68(63.0 %)	38(20.2 %)	0.462
Warfarin *n*, (%)	25(23.1 %)	50(26.6 %)	0.579
Death *n*, (%)	6(5.6 %)	15(8.0 %)	0.490
Admission SBP (mmHg)	155.0 ± 28.5	144.6 ± 29.7	0.003
Admission DBP (mmHg)	80.4 ± 15.2	78.3 ± 13.8	0.556
BMI (kg/m^2^)	23.5 ± 2.8	23.1 ± 2.6	0.354
HR (bpm)	90.0 ± 17.2	89.4 ± 19.0	0.677
HIF-1α(ng/ml)	2.70 ± 0.78	3.37 ± 0.79	0.001
NT-proBNP(ng/L)	6826.7 ± 7049.4	9297.7 ± 8359.8	0.007
TnT(ng/L)	85.5 ± 163.5	143.4 ± 241.1	0.015
hs-CRP (mg/l)	8.5 ± 5.7	9.2 ± 7.1	0.059
D-dimer (ng/ml)	294.4 ± 486.8	242.7 ± 312.3	0.302
Creatinine (ummol/l)	98.0 ± 45.4	100.8 ± 56.6	0.744
UA (ummol/l)	407.3 ± 137.4	389.2 ± 147.4	0.644
BUN(mmol/L)	10.2 ± 4.5	9.8 ± 5.1	0.521

*Abbreviations*: *SBP* systolic blood pressure, *DBP* diastolic blood pressure, *BNP* B-type natriuretic peptide, *hs-CRP* high sensitivity C-reactive protein, *cTNI* cardiac troponin I, *HR* heart rate, *TC* total cholesterol, *HDL* highdensity lipoprotein cholesterol, *LDL* low-density lipoprotein cholesterol, *UA* uric acid

**Fig. 1:**
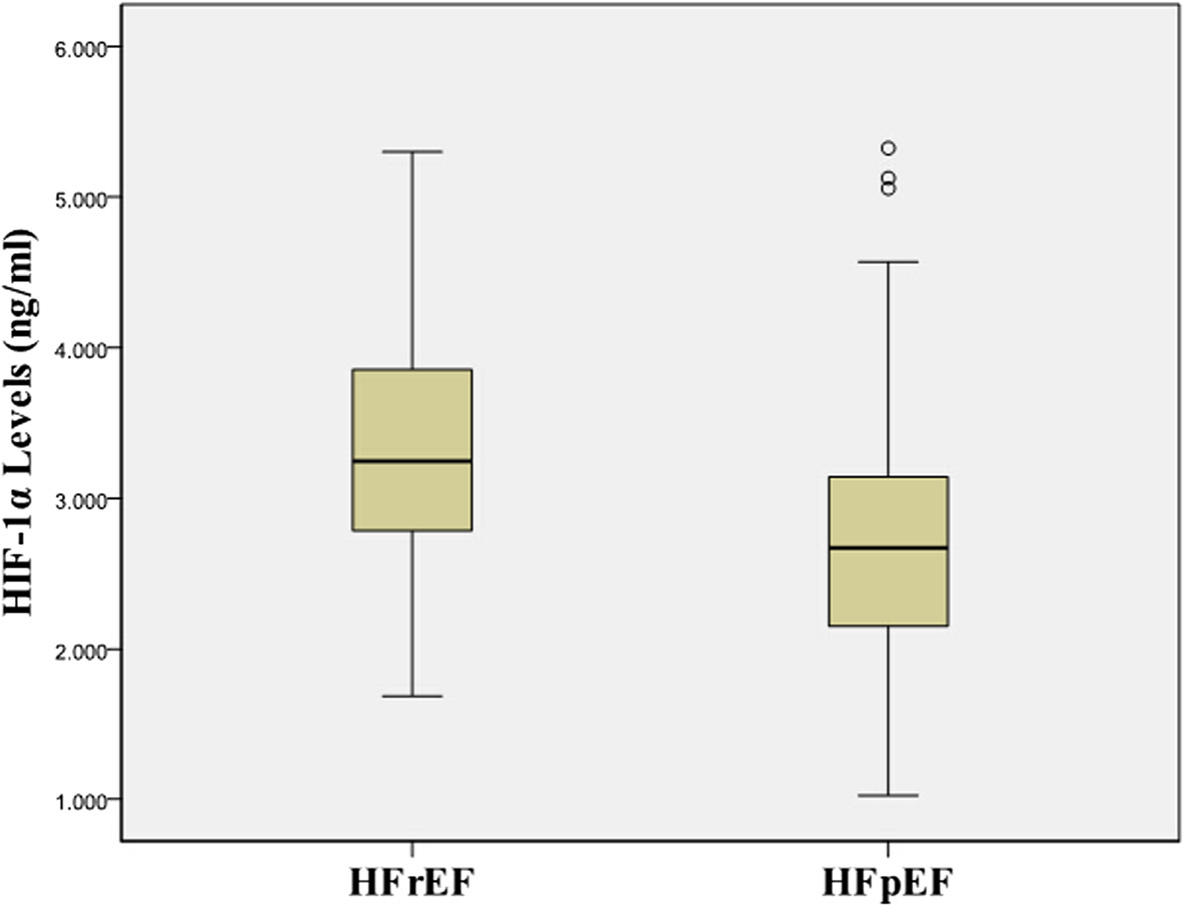
Comparison of HIF-1α values between HF*r*EF and HF*p*EF groups

**Fig. 2:**
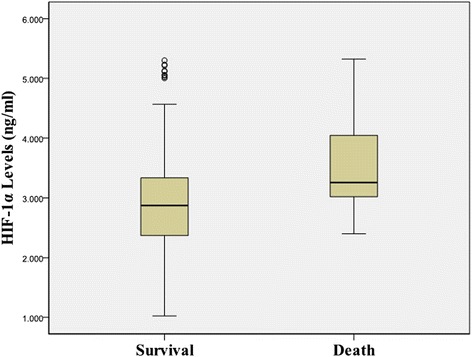
Comparison of HIF-1α values between survival and death groups

**Fig. 3:**
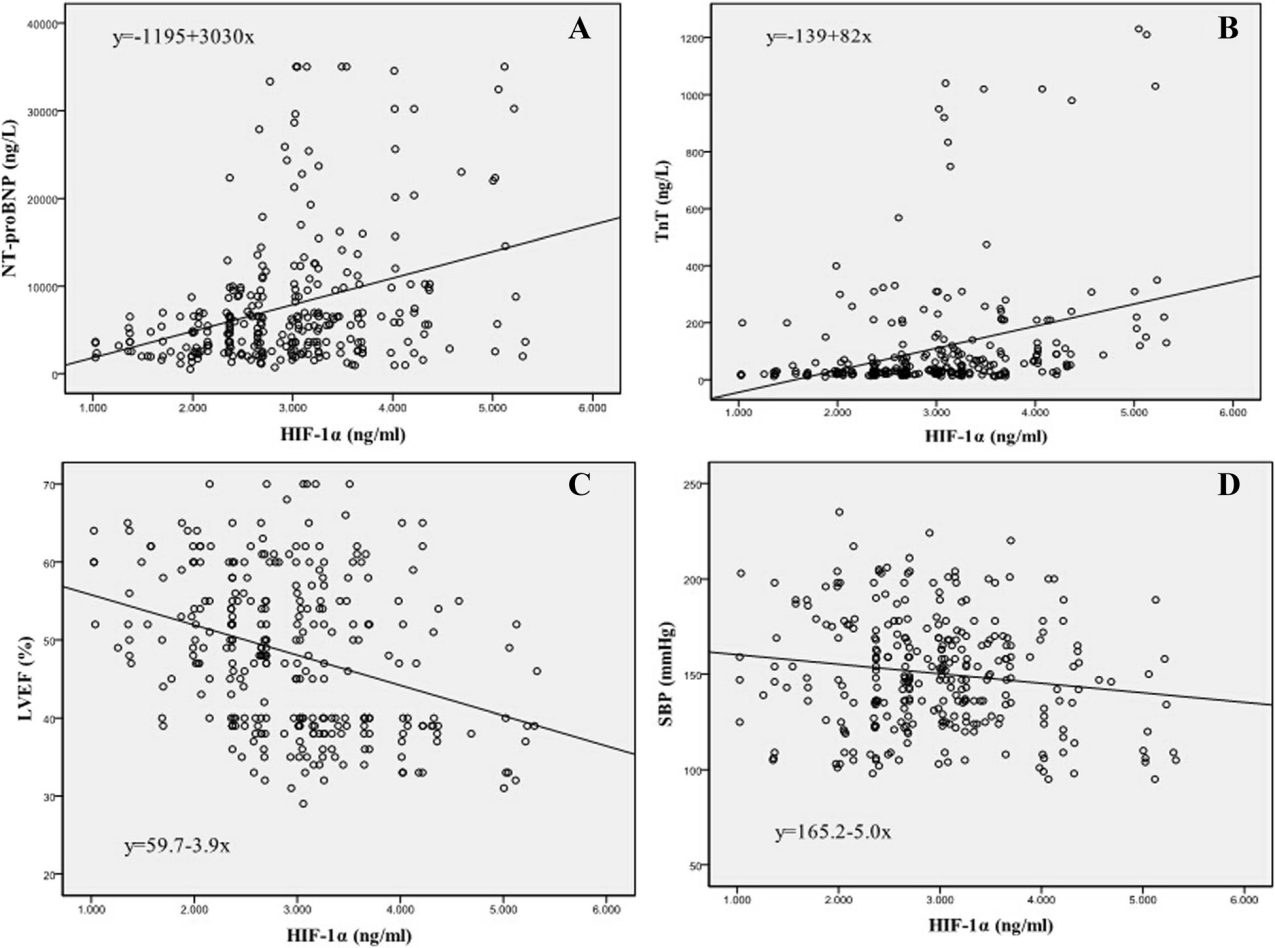
Scatter plots for the correlations between HIF-1α and NT-proBNP, TnT, LVEF and SBP

In our ADHF patients, the mean HIF-1α level was 2.95 ± 0.85 ng/ml and significantly higher than healthy subjects (1.31 ± 0.47 ng/ml, *p* < 0.001). However, serum HIF-1α levels in the HF*r*EF patients were significantly higher than the HF*p*EF patients (3.37 ± 0.79 *vs* 2.70 ± 0.78 ng/ml, *P* = 0.001, Table 1 and Fig. 1). During the study period, 21 patients died in hospital. In our findings, the HIF-1α concentrations were significantly higher in death than in survival patients (3.54 ± 0.81 *vs* 2.89 ± 0.83 ng/ml, *p* < 0.001, Fig. 2). HIF-1α levels positively correlated with NT-proBNP (*r* = 0.337, *P* < 0.001), TnT (*r* = 0.357, *P* < 0.001), and negatively correlated with LVEF (*r* = −0.332, *P* < 0.001) and SBP(*r* = −0.145, *P* = 0.013) but did not correlate with age, gender, hs-CRP, creatinine and HR (Fig. 3).

**Fig. 4:**
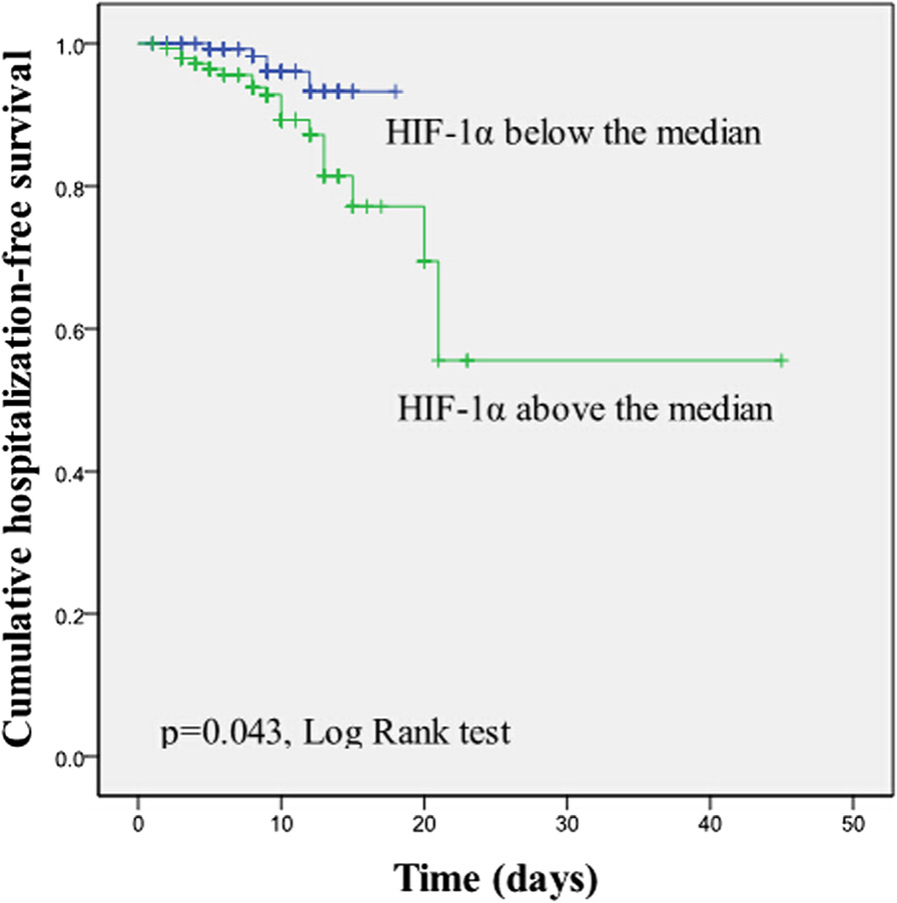
Cumulative hospitalization-free survival according to serum HIF-1α level (median: 2.95 ± 0.85 ng/ml)

In the present study the in-hospital mortality was 7.9 % (21 cases). The median hospital stay was 10.5 ± 8.9 days. Univariate Cox regression model results showed that the serum HIF-1α level predicts the risk of in-hospital mortality for ADHF patients (HR, 1.996; 95 % CI, 1.252–3.182, *P* = 0.004 [Table 2]); however, the final multivariate Cox regression model was performed using a stepwise method starting with variables that in univariate analysis were not associated with HIF-1α level and the risk of in-hospital mortality (Table 3). We dichotomized patients into two groups according to the HIF-1α median level. We found that the in-hospital mortality in the above median group was higher than the below median group (16 deaths vs. five deaths; 10.8 % vs. 3.4 %, *p* = 0.022). The Kaplan–Meier curves stratified according to the mean HIF-1α level are shown in Fig. 4. Log-rank testing revealed a significant increase in in-hospital mortality in the above median group as compared with the below median group (*p* = 0.043).

**Table 2:** Univariate Cox regression analysis for the identification of predictors of death

	OR	95 % CI	*P* Value
Age(years)	0.984	0.950–1.020	0.388
Male	0.992	0.407–2.415	0.985
History of hypertension	1.170	0.890–1.537	0.261
History of DM	1.252	0.481–3.258	0.645
Previous heart failure	1.425	1.119–4.449	0.115
Abnormal ECG	1.042	0.949–1.144	0.391
Admission SBP	1.003	0.989–1.017	0.711
Admission DBP	0.989	0.975–1.004	0.141
HR	0.980	0.957–1.003	0.093
Nitroglycerin	0.874	0.801–0.967	0.754
Digoxin	1.027	0.871–1.315	0.249
Diuretics	1.354	1.047–2.649	0.321
ACE inhibitors	1.144	0.977–2.042	0.219
Beta-blocker	0.907	0.865–1.141	0.476
HIF-1**α** (ng/ml)	1.996	1.252–3.182	0.004
Type of ADHF	3.946	1.613–9.652	0.003
LVEF	0.930	0.886–0.977	0.004
Maximal aortic diameter	1.037	0.997–1.079	0.073
TnT (ng/L)	1.270	0.223–7.220	0.788
hs-CRP (mg/l)	0.983	0.921–1.049	0.606
Creatinine (ummol/l)	0.993	0.979–1.007	0.342
BUN (mmol/L)	1.085	1.019–1.155	0.215
UA (ummol/l)	0.996	0.987–1.006	0.444

**Table 3:** HIF-1α levels predict the risk of in-hospital mortality for Cox regression model

HIF-1α	HR (95 % CI)	*P* Value
Unadjusted	1.996 (1.252–3.182)	0.004
Model 1	1.633 (0.999–2.672)	0.051
Model 2	1.724 (1.024–2.904)	0.040
Model 3	1.128 (0.594–2.140)	0.713

*Model 1* age, type of ADHF and sex, *Model 2* Model 1 + SBP, LVEF and HR, *Model 3* Model 2+ creatinine, hs-CRP, NT-proBNP and TnT

**Fig. 5:**
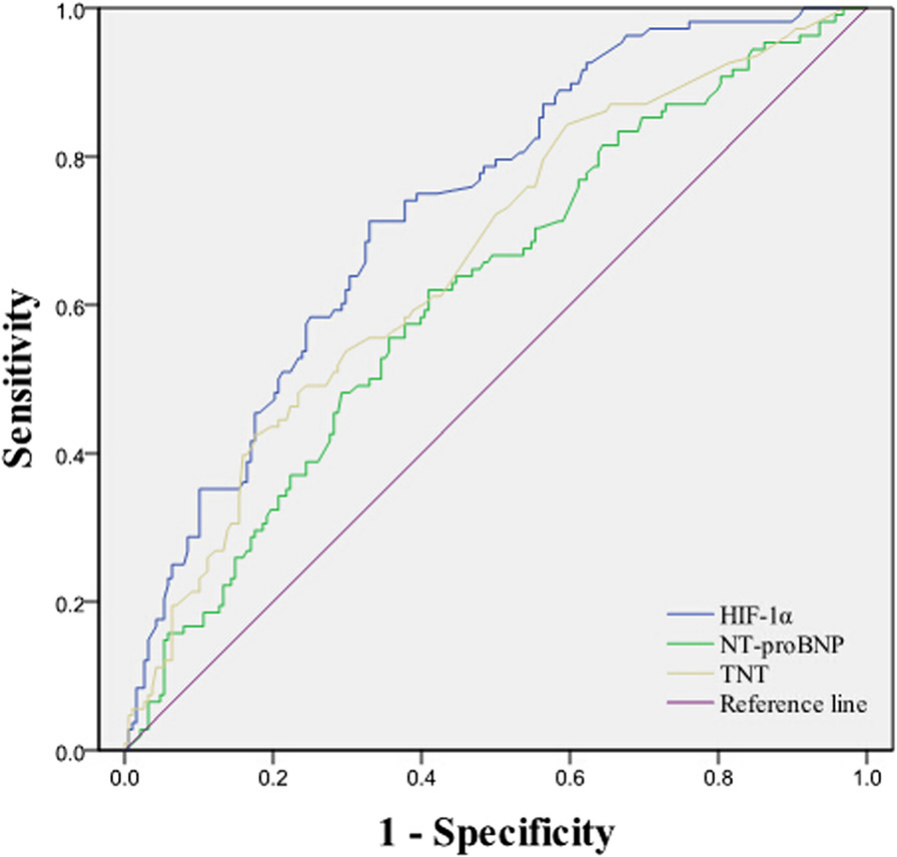
Diagnostic value of HIF-1α, TnT and NT-proBNP for type of ADHF

We performed receiver operating characteristic analysis to determine the cut-off value of HIF-1α, NT-proBNP and TnT in evaluating the type of ADHF of all patients. The best cut-off value with HIF-1α, NT-proBNP and TnT were 2.998 ng/ml (95 % CI: 2.357–2.998; sensitivity: 71.30 %; specificity: 67.02 %, *P* < 0.0001), 5573 ng/L (95 % CI: 2547–6587; sensitivity: 62.04 %; specificity: 59.04 %, *P* = 0.0005) and 86 ng/L (95 % CI: 47–180; sensitivity: 52.17 %; specificity: 74.73 %, *P* < 0.0001). The area under the curve were 0.730(95 % CI: 0.676–0.780, *P* < 0.0001), 0.617(95 % CI: 0.559 to 0.672, *P* = 0.0005) and 0.662(95 % CI: 0.605–0.715, *P* < 0.0001) for HIF-1α, NT-proBNP and TnT, respectively (Table 4 and Fig. 5).

**Table 4:** Diagnostic value of HIF-1α, TnT and NT-proBNP for type of ADHF

	AUC	Cut-off value	Sensitivity (%)	Specificity (%)	95 % CI	p Value
HIF-1α	0.730	3.62	35.2	90.0	0.676–0.780	<0.0001
TnT	0.662	0.12	42.6	82.5	0.598–0.725	<0.0001
NT-proBNP	0.502	19283	15.7	94.2	0.434–0.570	0.955

## Discussion

This is the first study to measure serum HIF-1α levels in ADHF patients. We confirmed that HIF-1α exists in the peripheral circulation of ADHF patients. The serum HIF-1α level was associated with NT-proBNP, TnT, and LVEF. The level of HIF-1α was elevated significantly in HF*r*EF and deceased patients compared with HF*p*EF and surviving patients. Kaplan–Meier curves revealed a significant increase in in-hospital mortality in ADHF patients with increased HIF-1α levels. Based on a univariate Cox regression model, there was an association between HIF-1α and the risk of in-hospital mortality; however, multivariate Cox regression analysis showed that HIF-1α cannot predict the short-term prognosis of ADHF patients.

Our study confirmed that the level of HIF-1α was elevated significantly in HF*r*EF and deceased patients compared with HF*p*EF and surviving patients. This finding reflects more serious hypoxia in HF*r*EF and deceased patients. The significant reduction in cardiac systolic function, as well as inadequate blood perfusion of various organs, eventually led to severe hypoxia and a disordered internal environment. The previous study showed that myocardial hypoxia leads to increased expression of HIF-1α [9, 29]. HIF-1α induces the transcriptional activity of the downstream regulatory target gene, BNP [20]. Another in vitro study result showed that hypoxia increases the synthesis of AC16 cells and secretion of BNP through a HIF-1α-independent mechanism [19]. The current study was the first to confirm that there is a significant correlation between HIF-1α and NT-proBNP in ADHF patients in vivo. Due to dysfunction of cardiac constriction, the levels of HIF-1α and NT-proBNP were increased synchronously in HF*r*EF patients. Receiver operating characteristic (ROC) curve analysis indicated the value of HIF-1α in predicting that the type of ADHF is superior to the NT-proBNP and TnT levels. These results provide evidence that HIF-1α is tightly associated with ADHF.

Oxygen balance plays an important role in maintaining internal environment homeostasis. The oxygen concentration of cells is controlled precisely. This ingenious balance can be destroyed by heart disease, cancer, cerebrovascular disease, and chronic obstructive pulmonary disease [30]. As a transcription factor, HIF-1 is a key factor in maintaining oxygen balance in the human body [7, 11, 30]. In hypoxic conditions, HIF-1 is widely expressed in histocytes, mediates the hypoxic response, and induces gene expression related to the hypoxic response.

HIF-1α is an intracellular protein. The current study first detected HIF-1α in the peripheral circulation of ADHF patients. Under normal oxygen conditions, HIF-1α is extremely unstable with a half-life of 10 min [31]; however, under hypoxic conditions, intracellular hydrolysis of protein is prevented because proline and asparagine residues in HIF-1α are not hydroxylated [32], which provides the possibility for intracellular to extracellular transfer. It has been reported that the level of expression of HIF-1 can be maintain and is not decreased under sustained hypoxic conditions [33]. In ADHF patients, the level of expression of HIF-1α increased greatly due to hypoxia and ischemia caused by hypoperfusion. In our previous study, the HIF-1α level in patients with type II diabetes mellitus and coronary calcifications was measured [34]. We presumed that circulating HIF-1α may be due to local cell apoptosis, which was induced by hypoxia. The potential underlying mechanism was likely high-mobility group box 1 (HMGB1), which reflects necrosis [35]. Under hypoxic conditions, HIF-1α initiates expression of TNF-α in cardiomyocytes [36]. We assumed that the increase in the circulating HIF-1α level reflects the severity of myocardial damage. Our findings indicate that there is a positive correlation between HIF-1α and TnT.

HIF-1α as a conditional knockout in myocardial cells will influence the maintenance of vascular endothelial growth factor (VEGF) expression [37] and angiogenesis [38]. It is essential for angiogenesis to increase oxidation transfer to compensate for hypertrophy. HIF-1α deficiency will accelerate the onset of heart failure in TAC after 3 weeks [12]. In pressure overload-induced heart failure, HIF-1α can protect heart failure [7]. The effect of HIF-1 has a complex pathophysiologic mechanism. During the development of cardiac hypertrophy, HIF-1 may have a protective effect and angiogenesis-promoting effect [39]; however, HIF-1 also has a pathogenic effect in terminal heart failure. As mediated by metabolism, heart failure is activated [7]. It has been reported that the cardiac function of HIF-1α+/− is damaged [40] or improved [6] relative to wild-type mice. These controversial results may reflect the complexity of the adaptive response mediated by HIF-1.

In our study population the univariate Cox regression model and Kaplan—Meier curve results showed that HIF-1α is a biomarker for predicting the risk of in-hospital mortality in ADHF patients; however, the final multivariate Cox regression model indicated that the HIF-1α level cannot predict the risk independently. Several hypotheses may explain this result. First, although HIF-1α can sustain the level of expression under hypoxic conditions, HIF-1α, as an intracellular protein in the peripheral circulation, may be not stable. Second, previous studies have shown that digitalis can inhibit the synthesis of HIF-1α [41]. In our patients, the administration of digitalis before admission and during hospitalization may affect the prognostic role of HIF-1α. Finally, oxygen inhalation during treatment in the hospital affects the expression of HIF-1α. With the improvement in the state of hypoxia, HIF-1α levels may decline rapidly. Overall, HIF-1α can provide an immediate reflection of tissue oxygenation at ADHF patients; however, we failed to demonstrate that HIF-1α is a biomarker for predicting the risk of ADHF.

### Study limitations

Our study had limitations. The sample scale and short follow-up time were disadvantages. Furthermore, to reduce patient discomfort, we did not perform arterial blood gas analysis. Moreover, we evaluated the degree of hypoxia in patients, so we did not detect HIF-1α mRNA levels. Finally, the present study was a multicenter study. Even though all patients had received standard treatment according to the guidelines during the hospital stay, there were some minor differences in treatment between each study center, such as oxygen concentration and flow rate, as well as the dose of diuretics. These differences may have resulted in statistical bias.

## Conclusion

The present study was the first to evaluate circulating levels of HIF-1α in ADHF patients. HIF-1α, as an intracellular protein, was originally shown to exist in the circulation of ADHF patients. Serum HIF-1α levels may reflect a serious state in patients with ADHF. Within the limitations of the study, serum HIF-1α levels were not correlated with patient outcome. The HIF-1α level may become a prognostic biomarker of heart failure; however; this potential role needs to be validated by means of further prospective studies in the future.

## Abbreviations

ADHF: Acute decompensated heart failure
ATP: Adenosine triphosphate
BNP: B-type natriuretic peptide
CHF: Chronic heart failure
TAC: Transaortic constriction
VEGF: Vascular endothelial growth factor
